# Concentration Effect over Thermoresponse Derived from Organometallic Compounds of Functionalized Poly(*N*-isopropylacrylamide-*co*-dopamine Methacrylamide)

**DOI:** 10.3390/polym13223921

**Published:** 2021-11-12

**Authors:** María Moral-Zamorano, Isabel Quijada-Garrido, Verónica San-Miguel, Berna Serrano, Juan Baselga, Saud Hashmi, Florian J. Stadler, Alberto García-Peñas

**Affiliations:** 1 Departamento de Ciencia e Ingeniería de Materiales e Ingeniería Química, IAAB, Universidad Carlos III de Madrid, Avda. de la Universidad, 30, 28911 Leganés, Madrid, Spain; mamoralz@ing.uc3m.es (M.M.-Z.); vmiguel@ing.uc3m.es (V.S.-M.); berna@ing.uc3m.es (B.S.); jbaselga@ing.uc3m.es (J.B.); 2 Instituto de Ciencia y Tecnología de Polímeros ICTP-CSIC, Juan de la Cierva 3, 28006 Madrid, Madrid, Spain; iquijada@ictp.csic.es; 3 Department of Polymer and Petrochemical Engineering, NED University of Engineering and Technology, University Road, Karachi 75270, Pakistan; saudhashmi@cloud.neduet.edu.pk; 4 College of Materials Science and Engineering, Shenzhen Key Laboratory of Polymer Science and Technology, Guangdong Research Center for Interfacial Engineering of Functional Materials, Nanshan District Key Laboratory for Biopolymers and Safety Evaluation, Shenzhen University, Shenzhen 518055, China

**Keywords:** thermoresponsive polymers, hydrophobic transitions, lower critical solution temperature, functionalized materials, contact angle

## Abstract

The functionalization of smart polymers is opening a new perspective in catalysis, drug carriers and biosensors, due to the fact that they can modulate the response regarding conventional devices. This smart response could be affected by the presence of organometallic complexes in terms of interactions which could affect the physical chemical properties. In this sense, the thermoresponsive behavior of copolymers based on *N*-isopropylacrylamide (NIPAM) could be affected due to the presence of hydrophobic groups and concentration effect. In this work, the functionalization of a copolymer based on NIPAM and dopamine methacrylamide with different amounts of bis(cyclopentadienyl)titanium (IV) dichloride was carried out. The resulting materials were characterized, showing a clear idea about the mechanism of functionalization through FTIR spectroscopy. The thermoresponsive behavior was also studied for various polymeric solutions in water by UV–vis spectroscopy and calorimetry. The hydrophobic interactions promoted by the organometallic complex could affect the transition associated with the lower critical solution temperature (LCST), specifically, the segments composed by pure NIPAM. That fact would explain the reduction of the width of the LCST-transition, contrary to what could be expected. In addition, the hydrophobicity was tested by the contact angle and also DNA interactions.

## 1. Introduction

The new strategies for sustainable progress developed by some governments are affecting industry, which needs to look for alternatives to traditional methods of production and also for new eco-friendly materials. Those changes must maintain companies’ profitability and provide other benefits such as improving the efficiency of industrial processes.

The generation of smart materials is a valuable way to respond to a part of industrial demand as they can improve the efficiency of some processes, reduce the consumption of energy, and are biodegradable in some cases where biodegradable monomers are involved as well [1,2,3,4,5,6,7,8,9,10,11,12,13,14,15]. Many efforts focus on the preparation and characterization of these materials, but the composites and functionalized polymers are growing as those can provide specific and modulated properties. In this sense, the preparation of smart polymeric matrices with inorganic compounds can provide additional functions to the pure polymers used in many applications [16,17,18]. Nevertheless, the problems associated with the lability of the metal–carbon bonds partially stopped this progress, but new regulation and advances in macromolecular chemistry have renewed interest in these structures [19,20]. 

There are some recent advances reported where smart polymers are involved in the preparation of hybrid materials for heterogeneous catalysis, which offer some advantages in terms of easy separations and recyclability [21,22,23]. However, applications can be extended to other fields as biomedical applications [8,24,25,26,27,28] or nanotechnology [29]. 

The final properties of the new materials will depend on the molecular details associated with the polymer and the organometallic complex, but also the relation between both will play an important role [30]. For example, a sufficiently high content of an organometallic complex could affect the physicochemical properties of the polymers. On the other hand, the functionalization of the smart polymers with organometallic complexes can be limited by different reasons like the numerous steps involved in the procedures, hazardous solvents, or the limitations for incorporating high amounts of an organometallic complex into the polymeric matrices, among other factors. 

The use of comonomers into the polymeric chains, as catechol groups, can provide other functionalities or reversibility. Recently, our research group proposed the novel and facile functionalization of the –OH groups of poly(*N*-isopropylacrylamide-*co*-dopamine methacrylamide), where we observed some particularities associated with the presence of the organometallic complexes [31]. Those copolymers were functionalized with an organotin (IV) compound and bis(cyclopentadienyl)titanium (IV) dichloride showing the hydrophobic interactions promoted by the organometallic complexes over the surrounding polymeric chains. Nevertheless, these preliminary works presented and analyzed functionalized materials with a minimum amount of metallodrugs as we wanted to preserve the original structure of the materials and also keep the thermoresponsive nature of the polymers [8,31]. In this sense, there are many questions that need to be addressed about the presence of organometallic complexes in smart polymeric chains, such as: How could affect the presence of the organometallic complex to the lower critical solution temperature (LCST) transition? What kind of interactions can be produced between polymeric chains and their hydrophobic units composed by the comonomer and organometallic complexes? Could the concentration of functionalized polymers in water change the LCST behavior due to the presence of surrounding polymeric chains?

This work tries to take a step towards answering these by working with different amounts of organometallic complexes over the same smart polymer, analyzing the changes promoted into the matrix and the thermoresponsive behavior of the corresponding polymeric solutions prepared with different concentrations in water. For that purpose, a copolymer based on *N*-isopropylacrylamide and dopamine methacrylamide was prepared and divided into different fractions. Each fraction of the copolymer was functionalized with a specific amount of bis(cyclopentadienyl)titanium (IV) dichloride, obtaining a wide range of different samples with different amounts of the organometallic complex. A complete characterization of the molecular features was carried out through proton nuclear magnetic resonance, Fourier-transform infrared spectroscopy, X-ray fluorescence, and X-ray diffraction. The thermoresponsive behavior was studied by differential scanning calorimetry and ultraviolet–visible (UV–vis) spectroscopy for both polymeric aqueous solutions (1 and 4 wt.%). The hydrophobicity was analyzed by the contact angle, and also, the DNA interactions were tested for the new materials.

## 2. Materials and Methods

### 2.1. Materials

*N*-isopropylacrylamide (NIPAM) (98%, Biosynth Carbosynth, Compton, UK) was purified by recrystallization using toluene (99%, Alfa Aesar, Haverhill, MA, USA) and hexane (96%, Scharlau, Sentmenat, Spain). Tetrahydrofuran (99.8%, Scharlau, Barcelona, Spain) and *N,N*-dimethylformamide (DMF) (99.8%, Alfa Aesar, Kandel, Germany) were dried before use. The azobisisobutyronitrile (AIBN) (98%, Sigma Aldrich, Saint-Quentin-Fallavier, France) was recrystallized from methanol (99.99%, Quimipur, Campo Real, Spain).

The preparation of the dopamine methacrylamide (DMA) involved the use of sodium borate (98%, Panreac, Barcelona, Spain), sodium bicarbonate (99%, Fluka, Hamburg, Germany), methacrylate anhydride (94%, Sigma Aldrich, Hamburg, Germany), 3,4-dihydroxyphenethylamine hydrochloride (98.5%, Fluka, Germany), sodium hydroxide (97%, Sigma Aldrich, Hamburg, Germany), ethyl acetate (99.91%, Quimipur, Campo Real, Spain), hydrochloric acid (37%, Sigma Aldrich, Hamburg, Germany), water (Quality Level: 200, Sigma Aldrich, Buchs, Switzerland) and magnesium sulfate (97%, Panreac, Europe Union).

The resulting polymer was precipitated in diethyl ether (99.7%, Quimipur, Campo Real, Spain). The functionalization was carried out using triethylamine (NEt3) (99%, Sigma Aldrich, Overijse, Belgium), and bis(cyclopentadienyl)titanium (IV) dichloride (also called titanocene dichloride, Cp_2_TiCl_2_) (97%, Sigma Aldrich, Moscow, Russia). The DNA interactions were performed with Buffer (Hyclone Products Cytiva, Amersham, UK) and salmon DNA (≤5% protein, Sigma Aldrich, Tokyo, Japan).

### 2.2. Synthesis of Dopamine Methacrylamide 

Dopamine methacrylamide (DMA) was prepared following the procedure previously published in the literature [32]. Subsequently, the DMA was purified by precipitation in hexane.

### 2.3. Preparation of Poly(N-Isopropylacrylamide-Co-Dopamine Methacrylamide) Copolymer 

The copolymer based on *N*-isopropylacrylamide and dopamine methacrylamide was synthesized using a Schlenk tube under a nitrogen atmosphere. For that purpose, the *N*-isopropylacrylamide (0.0663 mol), dopamine methacrylamide (0.0046 mol), and AIBN initiator (7 × 10^−5^ mol) were added inside the Schlenk tube. Then, 10 mL DMF was injected in the tube, keeping the inert conditions. The Schlenk tube was located inside of a thermostatic bath at 70 °C where the reaction took place for 48 h. After, the reaction was stopped using nitrogen liquid, the copolymer was precipitated in diethyl ether.

The final polymer was washed and purified several times through precipitation, dried under vacuum and, finally, it was stored at room temperature. The details about the synthetic procedure and reaction schemes can be found in previous works reported by the authors [31,33]. The yield of the reaction obtained was around 68%. 

### 2.4. Functionalization Process with Bis(cyclopentadienyl)titanium (IV) Dichloride 

The copolymer was divided into four parts which were functionalized with different amounts of bis(cyclopentadienyl)titanium (IV) dichloride. For that purpose, each part of the copolymer was independently dissolved in Schlenk tubes using DMF as the solvent and under nitrogen atmosphere. A solution of triethylamine (0.0359 mol·L^−1^) was prepared in advance, and specific amounts were introduced inside the Schlenk tubes where, previously, polymeric solutions were prepared, as shown in Table 1. Then, an organometallic complex solution (0.0161 mol·L^−1^) was injected in the previous tubes following the amounts described in Table 1, for obtaining materials with different concentrations of organometallic complex in a wide range of compositions. The reaction was carried out at room temperature for 36 h, and subsequently, the polymer was precipitated in diethyl ether, washed, and filtered under inert conditions. Finally, the samples were dried under vacuum at room temperature. 

From now on, the pristine copolymer is denoted as cDMA, adding f for functionalized samples (cDMAf) which bis(cyclopentadienyl)titanium (IV) dichloride (Cp_2_TiCl_2_) content varies and increases (indicated from 1 to 4) as collected in Table 1. 

### 2.5. Preparation of Polymeric Solutions 

The phase transition temperature associated with the LCST and the contact angle was studied for the pure copolymer and functionalized materials. For that purpose, two polymeric concentrations in water (1 and 4 wt.%) were prepared, depending on the technique and its resolution and for checking the differences associated with the interactions between polymeric chains in water. The solutions in water were kept in the refrigerator for homogenization of the samples for 24 h before measurements. 

All measurements were carried out at least in triplicate, which guarantees the consistency of these results.

### 2.6. Gel Permeation Chromatography 

Molecular weight distributions and polydispersity (M_w_/M_n_) were determined by a Waters SEC system (Milford, MA, USA) equipped with a Waters 1515 Isocratic HPLC Pump and a Waters 2414 refractive index detector, using DMF with LiBr 0.1 wt.% as the mobile phase at a flow rate of 0.7 mL·min^−1^ and 70 °C using polystyrene standards for the calibration.

### 2.7. X-ray Diffraction 

The measurements were carried out in a Philips X’Pert diffractometer provided with a PW3011/10 detector (Eindhoven, The Netherlands). The diffraction scans were collected between 5 and 80° with a 2θ step of 0.05° and 2.5 s per step.

### 2.8. Fourier Transform Infrared (FTIR) Spectroscopy

FTIR spectra were recorded by a Perkin-Elmer Spectrum Two FTIR-Spectrometer (Waltham, MA, USA) fitted with attenuated total reflectance (ATR). The samples were placed in direct contact with the diamond crystal without additional preparation. Measurements were collected from 4000 to 400 cm^−1^ at a resolution of 4 cm^−1^ with 32 scans per spectrum.

### 2.9. Nuclear Magnetic Resonance (^1^H NMR) Spectroscopy

^1^H-NMR spectra were recorded in deuterated DMSO at 400 MHz on a Bruker Avance III-HD 400 spectrometer (Billerica, MA, USA). 

### 2.10. X-ray Fluorescence Spectrometry 

The content of organometallics into polymeric chains was estimated by X-ray fluorescence spectrometer (Ametek Materials Analysis Division Spectro Xepos, Devon-Berwyn, USA), to detect the Ti content of the samples. 

### 2.11. Ultraviolet–Visible (UV-Vis) Spectroscopy 

Cloud point measurements were carried out in a Cary 3 BIO-Varian UV–Visible spectrophotometer (Palo Alto, CA, USA) equipped with a Peltier temperature control device. The temperature was raised from 12 to 45 °C at a heating rate of 1 °C·min^−1^. 

The estimation of the cloud points and phase transition temperatures were carried out as previously reported [34].

### 2.12. Differential Scanning Calorimetry

The polymeric solutions (around 15 mg) were placed in aluminum pans for studying the phase transition temperature associated with LCST by differential scanning calorimeter (UC3M: Mettler Toledo DSC822e, L’Hospitalet de Llobregat, Spain) equipped with a cooling system. All the tests were carried out between 0 and 45 °C, using a heating rate of 5 °C·min^−1^. The calorimetric curves were normalized using another experiment for pure water.

### 2.13. Contact Angle

Contact angles were measured using a Dataphysics Contact Angle System OCA Camera: Teli CCD Camera for all the samples at different temperatures (below and above LCST). Measurements were carried out in a range from 15 to 40 °C every 5 °C. Dry polymer films were prepared by drop-casting, placing 80 μL of an aqueous solution (2 wt.%) on a glass surface with a Teflon frame for keeping a fixed diameter of 1 cm.

Finally, 2 μL water droplets were dispensed onto the dried surface of the polymeric film. The average contact angle value was obtained from at least three measurements for each copolymer.

### 2.14. DNA Interactions

First, each functionalized copolymer (25 mg) was mixed with ethanol (25 mL) and phosphate buffer solution (25 mL) and was stored in a refrigerator (4 °C). On the other hand, several DNA solutions (0.02, 0.03, 0.04, 0.05, 0.06, and 0.08 mg/mL) were prepared. Then, polymeric solutions (3 mL) were mixed with each DNA-solutions (3 mL), and shaken at 35 °C (30 min) before measurements.

The analysis was carried out using an UV–vis spectrometer using a scanning wavelength from 800 nm to 200 nm at 35 °C. The samples were compared with pure DNA the absorbance of which is between 260 and 280 nm.

## 3. Results and Discussion

The molecular characterization was carried out for the pure copolymers and their corresponding functionalized samples. It was expected from synthetic and preparation routes to obtain different degrees of organometallic functionalization in a wide range of compositions. Therefore, the pure copolymer structure could suffer molecular changes as organometallic moieties are incorporated, varying the final spectrum of properties of functionalized materials. 

The average molecular weight (M_n_) and polydispersity of the pure copolymer, analyzed by gel permeation chromatography, were 42,000 g·mol^−1^ and 2.9, respectively. The relatively high polydispersity can be clearly explained by the free radical polymerization. The use of RAFT agents or catalysts was avoided as those could negatively affect biocompatibility. Also, these could disrupt the effect of the content of the organometallic complex over the molecular features and structure of the polymer, and consequently, on the thermoresponsive behavior and hydrophobicity of the samples. The comonomer content was estimated by ^1^H NMR following the procedure described in the literature, obtaining a DMA content of 5.3 mol % [35].

On the other hand, the DMA content of polymeric chains was estimated by comparing the protons of the benzene ring and the protons of the -CH- of the side chain of NIPAM obtained ^1^H NMR, as reported previously [31,33].

**Figure 1:**
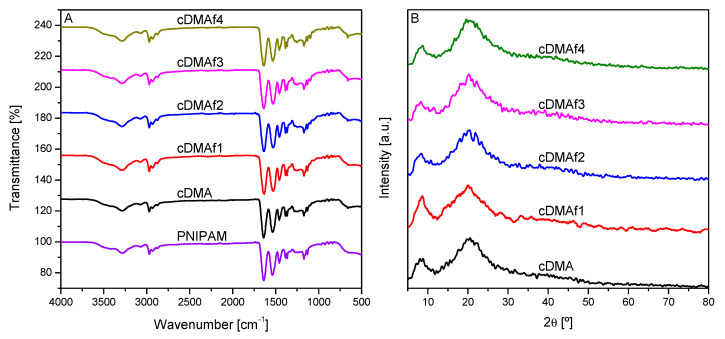
Microstructural features of the resulting materials characterized by Fourier transform infrared (FTIR) spectroscopy (**A**) and X-ray powder diffraction (**B**).

The molecular features of the resulting modified copolymers with Cp_2_TiCl_2_, and possible changes on the structures, were analyzed by FTIR spectroscopy, a powerful technique to elucidate interactions in organic-inorganic materials. FTIR spectra corresponding to PNIPAM, pristine cDMA, and copolymers functionalized with organometallic complexes are shown in Figure 1A. Differences between materials must be exclusively associated with the presence of organometallic complexes, as all the samples were prepared using the same copolymer composition. Typical vibration bands attributed to NIPAM and DMA appear in the spectra, at 3400–3300 cm^−1^ the O–H and N–H stretching, at 2970, 2930, and 2872 cm^−1^ the aliphatic C–H stretching, the strong bands (1640 and 1533 cm^−1^) corresponding to the C=O stretching of the amide (amide I) and N–H bending mixed with C–N stretching (amide II), at 1386 and 1366 cm^−1^ the typical doublet, corresponding to the deformation vibration of the isopropyl group in NIPAM. Significant changes are observed between 1000 and 1300 cm^−1^, specifically associated with the vibration bands observed at 1065 and 1253 cm^−1^, which are identified with ν(C–O) bonds related to catechol group. In this sense, the band placed at 1253 cm^−1^ is related to C–OH bonds, which intensity probably changes due to the functionalization of organometallic complexes through those -OH groups. Our previous works suggested this route of functionalization [8,31], but this is the first proof that could confirm that organometallic moieties are linked to the copolymer by the -OH groups. 

The FTIR spectroscopy can provide an idea about the degree of functionalization, which can be obtained through the band situated at 665 cm^−1^. That band is identified with δ(=C–H) bonds and is associated with the aromatic rings of Cp_2_TiCl_2_. The change of intensity could respond to the amount of organometallic complex incorporated into the polymeric chains.

X-ray diffraction patterns show exclusively two broad peaks, which are associated with the poly(*N*-isopropyl acrylamide) diffraction pattern (Figure 1B). [15,36,37] Differences between pristine copolymer and functionalized samples were not detected, even for the sample with the highest amount of Cp_2_TiCl_2_, suggesting that the organometallic complex was homogeneously distributed into the polymeric chains and did not lead to phase separation or crystallization; besides the concentration of organometallic complex does not affect the shape of the diffractogram probably because amounts are not high enough or the low resolution of the technique.

**Figure 2:**
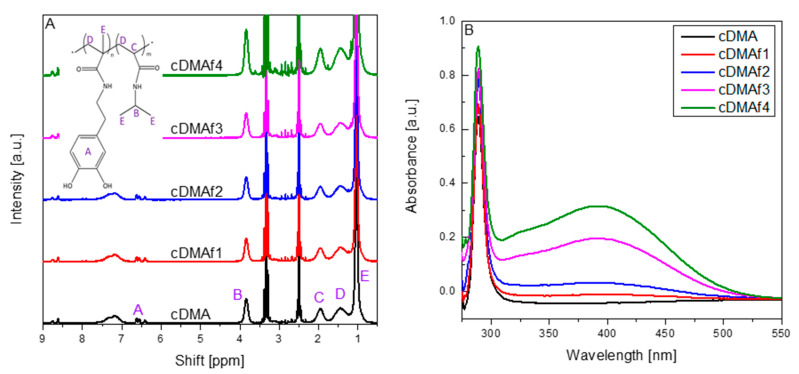
Molecular characterization for all the samples carried out by nuclear magnetic resonance (^1^H NMR) in DMSO-d6 (**A**), and ultraviolet–visible (UV–vis) spectroscopy (**B**).

The functionalization of the resulting polymers was also analyzed by ^1^H NMR, as Figure 2A displays. Significant changes cannot be detected in the NMR spectra as the organometallic compound content is very low compared to the total content of DMA inserted along the polymeric chains. Another important problem is related to the overlapping between proton signals corresponding to Cp_2_TiCl_2_ and those of the pure copolymer, as can be deduced from their respective spectra. Due to the interaction of DMA with the organometallic compound, we would expect to observe specific changes in the peaks placed at 8.6 and 8.75 ppm, associated with the protons of -OH groups. [31] Nevertheless, the low content of the organometallic complex with respect to the DMA units inserted along the polymeric chains could explain, together with the overlapping with the main chain peaks, the lack of changes in the spectra. The similarity between the different spectra seems to indicate that structural changes are avoided.

The functionalization of the samples was studied by UV–vis spectroscopy in aqueous solutions at ambient temperature as Figure 2B shows. The pure copolymer shows exclusively the absorbance of the C=O bonds, which are part of the NIPAM and DMA structure, but a second peak is identified for the functionalized samples. That second absorbance peak could be associated with the cyclopentadienyl groups and Ti-O bonds [38]. Furthermore, the concentration of organometallic compounds along the polymeric chains seems to play an important role, too, as the intensity of the peak increases as Cp_2_TiCl_2_ rises.

**Scheme 1:**
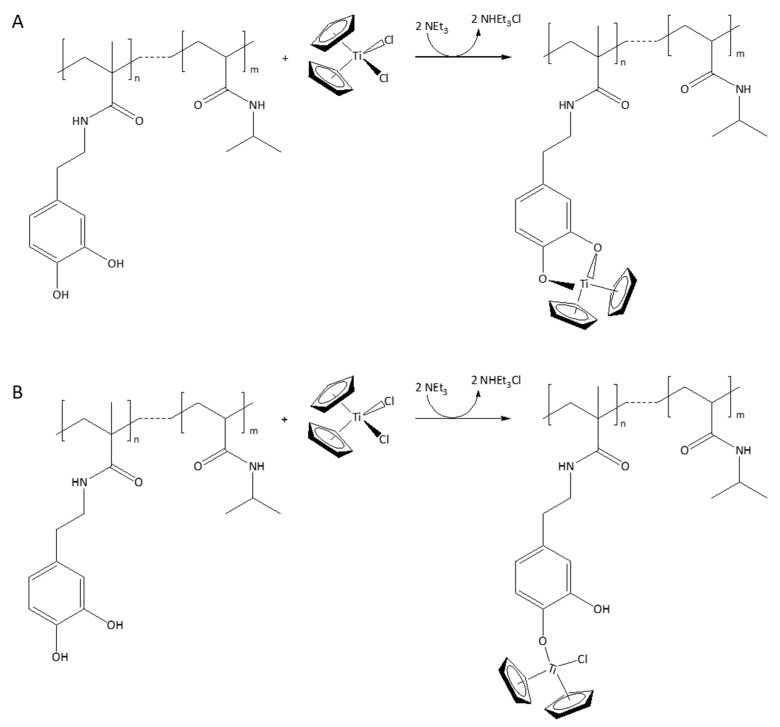
Possible routes of functionalization between copolymer and organometallic complex through both OH groups (**A**) or a single OH (**B**).

The elemental analysis was carried out by X-ray fluorescence for all the functionalized materials (Table 2). The percentage of Ti increases as was expected during the experimental work, showing the successful incorporation of organometallic compounds along the polymeric chains. Another important piece of information derived from elemental analysis is the relationship between Ti and Cl, which shows a value higher than 1 for cDMAf3 and cDMAf4. There are two possible routes for functionalization, i.e., this one may be carried out by the reaction of a single or both chlorine ligands through both OH-groups attached to the DMA (Scheme 1). The ratio between Ti and Cl seems to explain that a great part of the organometallic moieties is attached by a single bond to the polymeric structure as they seem to keep another chlorine (Scheme 1B). Nevertheless, these results are not conclusive, and the error associated with the measurements should be considered because it could play an important role.

**Figure 3:**
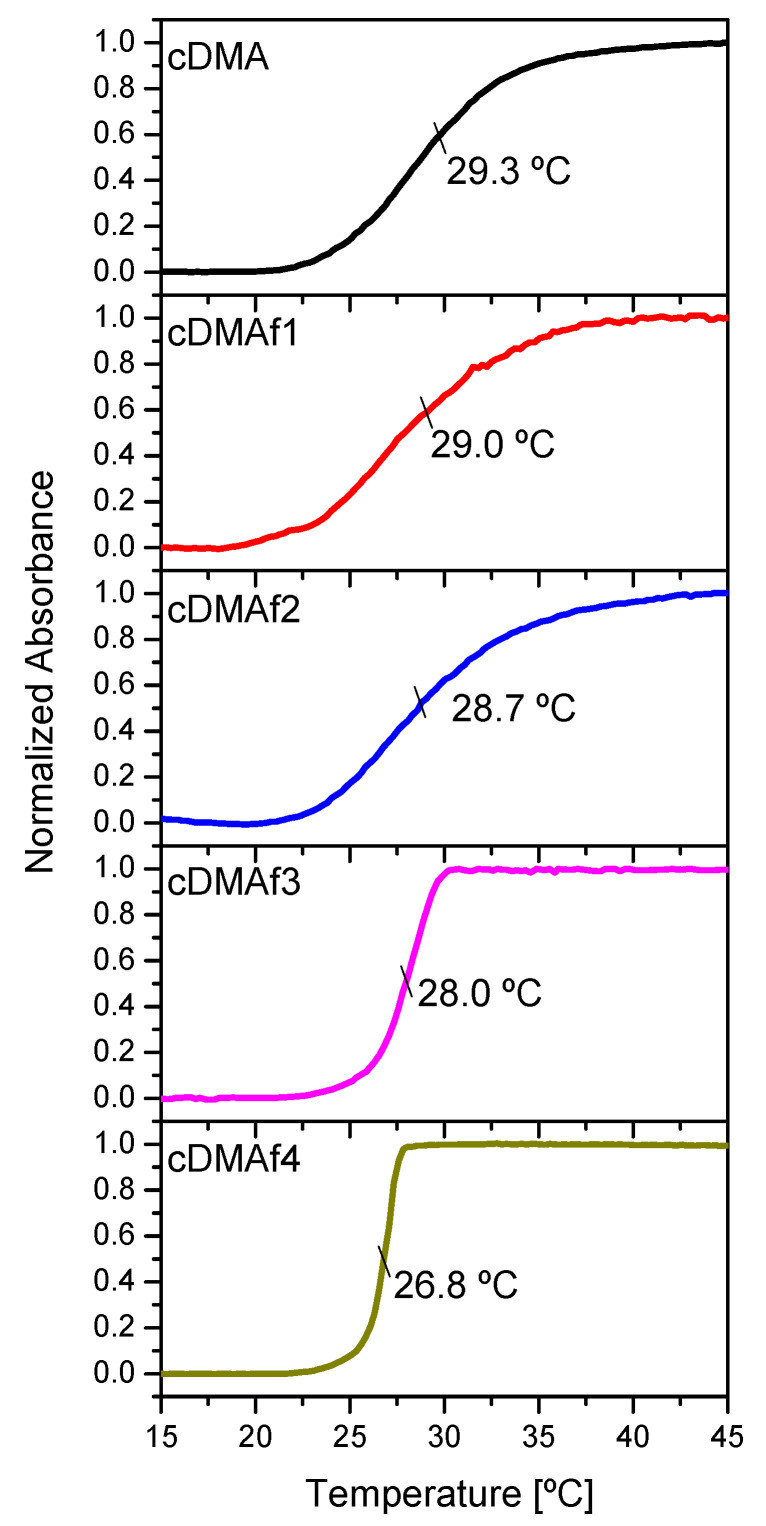
Phase transition temperatures, analyzed by UV–vis spectroscopy for all polymeric solutions (4 wt.%).

The thermoresponsive properties in aqueous solutions were studied for all the functionalized samples and the pure copolymer by UV–vis spectroscopy. Figure 3 displays the phase transitions associated with the LCST for polymeric solutions (4 wt.%), showing the transitions pretty close to each other. Nevertheless, significant differences can be observed from the shape of the curves. In general, a higher number of hydrophobic groups promotes broader transitions [33]. The explanation related to these broad hydrophobic transitions was reported before for similar samples, but the amount of comonomer was varied, keeping the same amount of the organometallic complexes [31]. In that case, rheology showed that the phase transition temperature associated with LCST comprises two partially overlapped transitions. One of them is related to NIPAM sequences free of DMA placed at the highest temperature, and another one is situated at a lower temperature associated with the segments of the chains with the presence of DMA. Higher content of DMA into the polymeric chains will induce broader transitions as the lowest transition temperature associated with segments with comonomer will be placed at a lower temperature, increasing its distance with the phase transition temperature of pure NIPAM sequences.

This explanation cannot apply to the content of organometallic complexes for these samples with the same composition of DMA as it can be observed the opposite effect, i.e., transitions are wider as the content of hydrophobic organometallic complexes rises. It could be feasible to think about similar effects as both comonomer and organometallic complexes present a hydrophobic nature. Nevertheless, the situation is somewhat different as expected and, consequently, some parameters such as polymer concentration in water, interactions between surrounding chains, organometallic complex amount, and position of the organometallic moieties into the polymeric chains could play an important role.

**Scheme 2:**
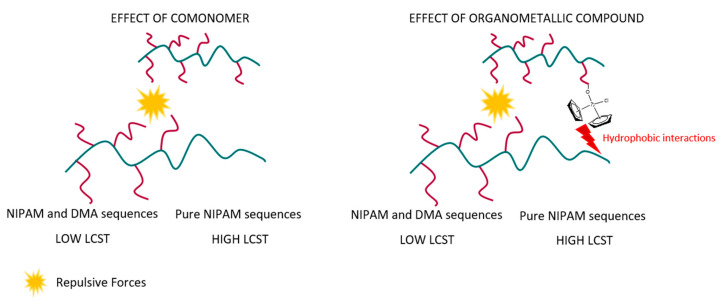
Effect of hydrophobic groups over surrounding polymeric chains in solution.

The polymer concentration in water could be high enough to allow interactions between polymeric chains which could be restricted by repulsive forces between DMA units and organometallic moieties. Nevertheless, a hydrophobic influence could be exerted by the organometallic complex over sections of surrounding polymeric chains composed by pure NIPAM. This influence could reduce the phase transition temperatures of LCST associated with these pure NIPAM sequences. Scheme 2 shows a clear idea about the differences between both effects. In the left part of the figure, the comonomer effect clearly will reduce the low phase transition temperature associated with the NIPAM + DMA sequences as comonomer content rises. The comonomer content will not affect the LCST of the NIPAM sequences, as they could not reach the main backbone due to repulsive forces between surrounding polymeric chains. This will lead to an increase in the width of the transition as the lowest LCST (NIPAM + DMA sequences) is reduced and the distance with the highest LCST (NIPAM sequences) increases.

The situation is rather different when the content of the organometallic complex rises to keep the same comonomer composition (right part of Scheme 2). The lowest LCST (NIPAM + DMA sequences) would be affected by organometallic compounds due to the repulsive forces between organometallic moieties and DMA units of surrounding polymeric chains that could protect part of the hydrogen bonds, increasing the lowest LCST. Those normally could be affected by the units of DMA of surrounding polymeric chains reducing the LCST. The content of organometallic complexes is too low compared to the comonomer content, but enough to partially reduce those interactions as organometallic compounds are too far from the main backbone. On the other hand, these organometallic compounds could probably affect pure NIPAM sequences of surrounding polymeric chains, decreasing the highest LCST, consequently inducing shorter hydrophobic transitions.

The cloud points were estimated for all the curves, and those decrease as the content of organometallic compounds rises, as expected. The hydrophobic groups could reduce the LCST, but this assumption needs to be studied in depth as molecular features of the polymeric chain in terms of composition, position, and distributions play an important role in the LCST mechanism.

**Figure 4:**
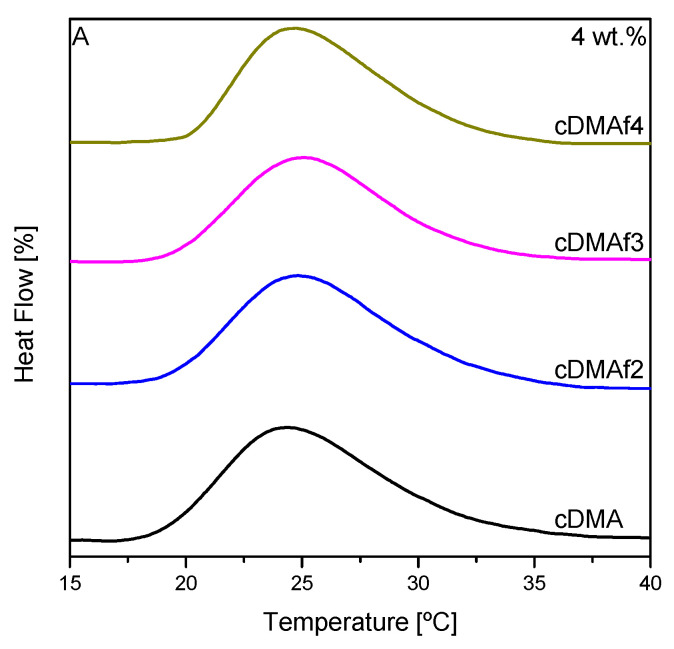

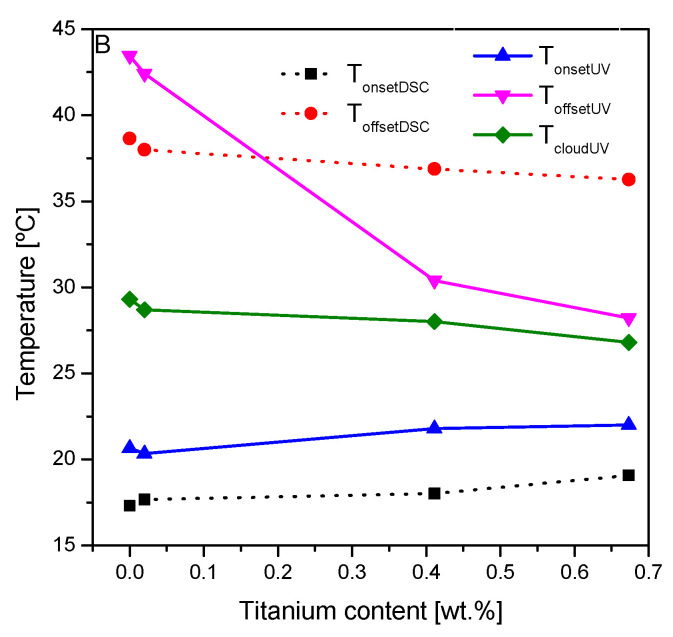
Phase transition temperatures associated with lower critical solution temperature (LCST), analyzed by calorimetry (**A**), and a comparison of calorimetric and UV results (**B**) for all polymeric solutions (4 wt.%).

Similar experiments were carried out by differential scanning calorimetry using the same polymer concentration in water but with higher heating rates because temperature control in a calorimeter is possible up to significantly higher rates than in optical cuvettes. These curves are displayed in Figure 4A, and it can be observed that the width of the hydrophobic transitions decreased as the content of organometallic complexes rose. The width of the transitions follows the trend observed by turbidimetry in Figure 3 but the maximum of the transitions does not look like to follow a specific tendency with the presence and the content of Cp_2_TiCl_2_. Turbidimetry showed that the cloud temperatures decreased as the organometallic complexes increased, but in this case, that behavior is not unambiguous. The differences between techniques could explain these changes, as UV–vis spectroscopy shows the reduction of the transmittance as increasing the phase separation structures, but calorimetry is very sensitive to the detection of the coil to globule transitions [34,39]. Many factors could explain that a clear tendency of the maximum of DSC is not exhibited, as end-group effect or the polymer–solvent interactions, which could be affected by the presence of the organometallic compound [40,41,42,43]. The reproducibility of the experiments confirms the results, and consequently, different phenomena should be responsible for this kind of curve.

Figure 4B shows the LCST transitions analyzed from curves of UV–vis spectroscopy and calorimetry. For that purpose, the onset and offset temperatures (T_onset_ and T_offset_) were defined where the transitions or LCST-ranges start and finish, respectively. Also, cloud points were included, which were estimated at 50% of the UV–vis transition [33]. The transitions clearly diminish their width as the content of the organometallic complex increases, i.e., onset and offset temperatures are closer to each other. Another important fact can be deduced from the cloud temperatures, whose tendency is defined by the content of the organometallic complex but clearly defined in the middle of the onset and offset temperatures estimated from DSC. In general, the UV transitions are shorter than those determined by calorimetry, as could be expected due to calorimetry being more sensitive [34].

T_offsetUV_ seems clearly affected in a higher proportion than T_onsetDSC_ by the content of the organometallic complex due to T_offsetUV_ being closer to the cloud points. T_offsetDSC_ and T_onsetDSC_ are defined proportionally at similar distances of the cloud points.

A small protection of a small part of the hydrogen bonds derived from the repulsive forces exerted between the organometallic moieties and the DMA of surrounding polymeric chains could explain the increase of the T_onsetUV_ and T_onsetDSC_, as explained above. On the other hand, the hydrophobic interactions promoted by the organometallic compounds over the pure NIPAM-sequences of the polymeric chains could also explain the decrease of the T_offsetUV_ and T_offsetDSC_.

**Figure 5:**
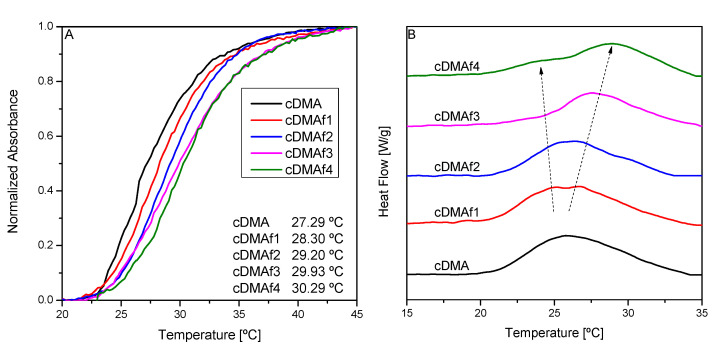
Phase transition temperatures analyzed by UV–vis spectroscopy (**A**) and calorimetry (**B**) for all polymeric solutions (1 wt.%).

There are many factors involved in the LCST. More information could be obtained if the polymeric interactions between different chains were diminished, i.e., if the polymer concentration in water decreased. For that purpose, the concentration of the polymeric solutions was decreased to 1 wt.%. The idea was to observe if there were noticeable changes when the interactions between polymeric chains were reduced. Figure 5A shows the phase transitions of LCST for all the polymeric solutions (1 wt.%) whose shape is similar for all the samples. In this sense, hydrophobic transitions do not show differences probably because interactions between different polymeric chains are minimized.

Another important fact is associated with the trend drawn by the cloud points, which increase as organometallic moieties rise. The reproducibility of the experiments repeated three times only opens the possibility of stretching of the polymeric chain due to the hydrophobic forces of the organometallic complexes. However, for a mechanistic discussion, a much wider range of samples would be necessary.

**Scheme 3:**
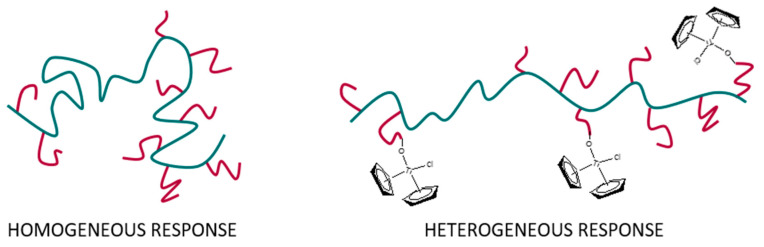
Homogeneous and heterogeneous LCST-response as a consequence of the incorporation of an organometallic compound.

Calorimetry could be a good tool for getting more information about these transitions as it is more sensitive than UV–vis spectroscopy. Figure 5B displays the calorimetric curves measured using a polymeric concentration in water of 1 wt.%. The pure copolymer exhibits a single transition which is subsequently divided into two parts as the content of the organometallic complexes rises. Both transitions are clearly distinguished for cDMAf3 and cDMAf4, showing that two phenomena are taking place. The effect could be associated with a stretching of the polymeric chains due to the presence of the organometallic compounds whose hydrophobic strength promoted by their length and volume over the main backbone could isolate the different polymeric sequences. Thus, this arrangement of the polymeric chains could induce a heterogeneous LCST-response regarding the pure copolymer where there are some interactions between the same polymeric chains (Scheme 3).

Transitions seem to be composed of the overlapping of two phenomena. The phenomenon or transition placed at lower temperature seems to decrease as the organometallic compound rises, while the highest one exhibits the opposite behavior, i.e., the peaks look displaced to higher temperatures. The effect at a lower temperature is clearly observed for the highest content of organometallic compound (cDMAf4), and could be related to the segments of the chains enriched in DMA and, consequently, in organometallic moieties. Then, it could be justified that the presence of these hydrophobic groups could move the transition to lower temperatures depending on the content of organometallics. Nevertheless, the second transition seems to follow the opposite trend, i.e., it increases as hydrophobic groups rise, probably due to the stretching of the polymeric chain as interactions with surrounding polymeric chains must be reduced.

**Figure 6:**
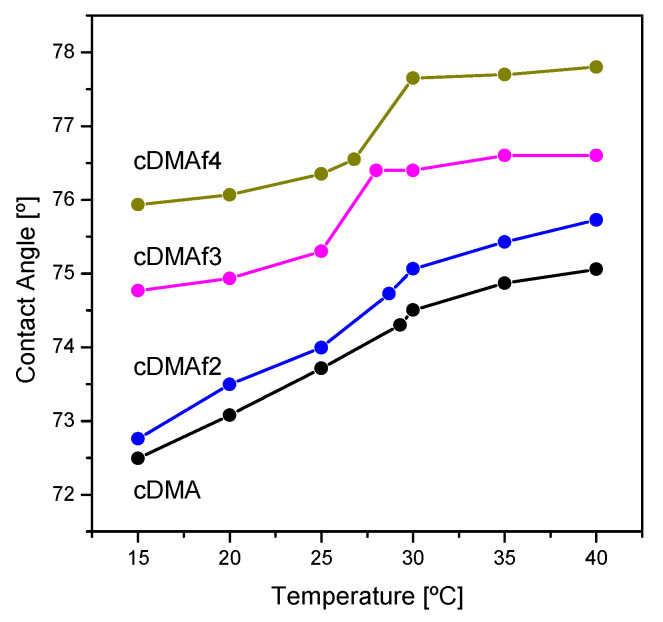
Contact angle values for functionalized samples and pure copolymer measured at different temperatures.

Figure 6 shows experiments related to the wettability of the functionalized structures and also for the pure copolymer measured at different temperatures below and above of LCST. Specifically, the hydrophobicity on the surface was evaluated by the water contact angle, where important differences can be observed. First of all, a clear trend is defined by the content of the organometallic complex, which induces a higher hydrophobicity inducing higher angles. Similar behavior was observed for other samples where hydrophilic or/and hydrophobic groups were involved. For PNIPAM an increase in the hydrophobicity was reported, while a copolymer with hydrophilic monomer decreases the contact angle [44]. On the other hand, PNIPAM-modified styrene-butadiene rubber was also studied in terms of contact angle for different contents of NIPAM, where it was demonstrated how strongly the hydrophilic behavior could be influenced below the LCST [45].

Specifically, cDMAf3 and cDMAf4 exhibit a clear jump around the LCST, which cannot be clearly detected for the pure copolymer or sample with a low amount of organometallic complex (cDMAf2). Nevertheless, these samples show a greater change between 15 °C and 40 °C as can be deduced from cDMAf2 where the angle increases around 3° while it is below 2° for cDMAf4. This fact could be associated with the presence of the organometallic complex, which could reduce the impact of the hydrophobic contributions of DMA below LCST as was observed for the hydrophobic transitions observed by calorimetry and UV–vis spectroscopy. The results fit well with the literature as remarkable changes are exclusively observed below LCST, while above the LCST, the angle remains constant [45].

The presence of the organometallic compound could play an important role along the transition LCST showing a clear jump which could be associated with the coil to globule transition where the organometallic moieties could be exposed outside of the globule due to its hydrophobic nature inducing those high angles. Nevertheless, this is a hypothesis, as the polymers are not dissolved in water.

**Figure 7:**
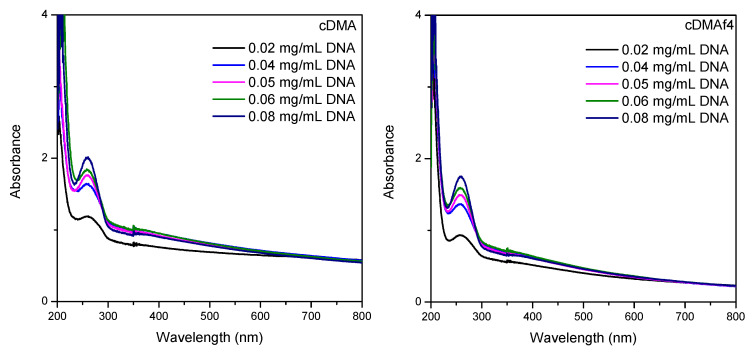
UV–vis spectra of DNA-interactions for cDMA (**left**) and cDMAf4 (**right**).

Figure 7 exhibits the absorbance of the suspensions composed of different concentrations of DNA and functionalized materials (cDMA and cDMAf4). For both samples, the intensity of the peaks rises when DNA concentration increases. In addition, a hypsochromic effect was observed between the different DNA concentrations for each sample, as was reported previously [31].

Both samples show the same trends, but it is important to indicate a change between the kind of peaks for the same DNA concentration but different samples. The peaks related to pure copolymer are slightly wider than for the functionalized sample cDMAf4, and consequently, this fact could be associated with the presence of the organometallic complex. Nevertheless, the experimental error could also be involved in these trends due to the low concentration of the organometallic complex.

## 4. Conclusions

The functionalization of copolymers based on N-isopropylacrylamide and dopamine methacrylamide allows high amounts of organometallic moieties incorporated in the polymeric chains to be obtained in comparison with other complex methods reported in the literature.

The functionalization of the polymers is carried out through the -OH groups, confirming the hypothesis reported in our previous publications about the functionalization routes. In this sense, the new copolymers allow getting information from FTIR spectroscopy which shows clear changes associated with ν(C–O) bonds in the band placed at 1253 cm^−1^. The incorporation of an organometallic complex can affect the LCST behavior, but the polymer concentration in water will define those changes as interactions with surrounding polymeric chains will be modified; if there are polymeric interactions between different chains, those will be influenced by the presence and content of an organometallic complex. Those could affect the polymeric chains composed of pure NIPAM reducing its phase transition temperature associated with the LCST for these polymeric sequences and, consequently, obtaining a shorter hydrophobic LCST-transition. If the interactions between surrounding polymeric chains are minimized (low polymeric concentrations in water), the organometallic complex could reduce the interactions between parts of the same polymer chain. This fact could open a new perspective concerning controlling the LCST transition due to the presence and the content of organometallic complexes.

Contact angle shows the effect of the incorporation of an organometallic complex into the polymeric chains due to its promotion of a higher hydrophobicity. In addition, these contributions seem to modulate the change of temperature below and above LCST.

The DNA-interactions were tested for the resulting materials, showing that DNA can be affected probably due to electrostatic interactions.

## Figures and Tables

**Table 1:** Functionalization conditions for copolymers functionalized with the organometallic complex (cDMAf1-4).

Sample	DMF [mL]	NEt3 [mol]	Cp_2_TiCl_2_/cDMA [mole Ratio]
cDMA	10	0	0
cDMAf1	10	2.03 × 10^−6^	2.43 × 10^−4^
cDMAf2	10	4.06 × 10^−6^	4.87 × 10^−4^
cDMAf3	10	2.03 × 10^−5^	2.43 × 10^−3^
cDMAf4	10	4.06 × 10^−5^	4.87 × 10^−3^

**Table 2:** Percentage of Ti and Cl incorporated into the copolymers determined by X-ray fluorescence (wt.%).

Sample	Cl [%]	Error	Ti [%]	Error
**cDMAf1**	0.0029	0.00001	0.00049	0.00001
**cDMAf2**	0.0590	0.00010	0.02013	0.00017
**cDMAf3**	0.3543	0.00030	0.41120	0.00090
**cDMAf4**	0.5165	0.00040	0.67320	0.00120

## Data Availability

Not applicable.

## Abbreviations

LCST	Lower critical solution temperature
NIPAM	*N*-isopropylacrylamide
DMA	Dopamine methacrylamide
Cp_2_TiCl_2_	bis(cyclopentadienyl)titanium (IV) dichloride
